# Physical frailty and its associated factors among elderly nursing home residents in China

**DOI:** 10.1186/s12877-020-01695-5

**Published:** 2020-08-17

**Authors:** Weiwei Liu, Martine Puts, Fen Jiang, Chuyi Zhou, Siyuan Tang, Sanmei Chen

**Affiliations:** 1 Xiangya Nursing School of Central South University, Changsha, Hunan China; 2 Lawrence S. Bloomberg Faculty of Nursing, University of Toronto, Toronto, Ontario Canada; 3 Medical College of Hunan Normal University, Changsha, Hunan China; 4 Department of Epidemiology and Public Health, Graduate School of Medical Sciences, Kyushu University, Fukuoka, Japan

**Keywords:** Frailty, Nursing home, Older adults, China, Aging

## Abstract

**Background:**

Evidence is scarce on the trend in prevalence of physical frailty in China; the primary purpose of this study was to identify the prevalence and correlates of physical frailty among older nursing home residents in China.

**Methods:**

Cross-sectional study in 20 nursing homes in Changsha, China. Physical frailty was defined based on the frailty phenotype including weight loss, low grip strength, exhaustion, slow gait speed, and low physical activity. Participants with at least three affected criteria were defined as being frail. Participants with one or two affected criteria were considered as pre-frail, and those with no affected criteria were considered as robust. A total of 1004 nursing home residents aged 60 and over were included in this study. A multinomial logistic regression model was used to analyze the associations of physical frailty with its potential risk factors, including age, sex, education levels, marital status, type of institution, living status, current drinking, current smoking, regular exercise, and self-reported health.

**Results:**

The overall prevalence of physical frailty and prefrailty was 55.6, and 38.5%, respectively. The rate of physical frailty substantially increased with age, and was higher in women than in men (69.5% vs. 30.5%). The multinomial logistic regression analysis showed that older age, being women, living in a private institution, living alone or with unknown person, having no regular exercise (≤ 2 times/week), and poor self-reported health were significantly associated with increased odds of being physically frail.

**Conclusion:**

We demonstrated physical frailty is highly prevalent among older residents in nursing homes in China, especially in women. The potential role of those associated factors of physical frailty warrant further investigations to explore their clinical application among elderly nursing home residents.

## Background

There is a growing research interest in frailty worldwide, especially in countries where the population is rapidly aging, including China [1, 2]. Frailty refers to a state of increased vulnerability to stressors, characterized by a decreased physiological reserves [3, 4], resulting in an elevated risk of adverse health outcomes, such as falls, disability, hospitalization, delirium and mortality [5, 6]. Although there is no universal consensus in the operational criteria used in different practice settings and epidemiological investigations [7], two main operational approaches have been widely used to measure frailty: the frailty phenotype [5] and the Frailty Index [8]. The Frailty Index is composed of at least 30 Items and can be obtained through a comprehensive geriatric assessment [3]. The frailty phenotype can be calculated by having older adults complete simple tasks without a preliminary clinical evaluation. The frailty phenotype is more easily identified objectively in older nursing homes residents who are at increased risk of negative events [9]. However, due to the differences (i.e., physiological and psychological dimension) in different populations, there are no reference criteria for the frailty phenotype in older adults living in nursing homes in China.

According to various previous studies among community-dwelling older adults in Western countries, the prevalence of frailty varied enormously (range 4.0 to 59.1%), which is likely due to different measurement tools and frailty definitions used [10]. The prevalence of frailty has been reported to vary between 5.9 to 17.4% in China [11]. For residents living in nursing homes, physical frailty is highly prevalent (range 19.0 to 75.6%) in western populations [12]. Numerous studies on frailty in China, to date, have been conducted in community-dwelling older adults [11], but epidemiological data is scant among older nursing home residents [13]. Compared to community-dwelling older adults, individuals living in nursing homes might be more vulnerable and tend to simultaneously have multiple risk factors of frailty (i.e., self-reported health status, activity of daily living disability) [14–16]; consequently, the prevention and management of frailty could be more challenging in nursing homes. Therefore, it is of substantial clinical interest to identify the prevalence of physical frailty and its modifiable risk factors among elderly nursing home residents. Such epidemiological evidence could help to develop effective interventions for the prevention and management of physical frailty in the nursing homes setting to delay the onset of frailty and maintain independence in daily activities [17–19]. Thus, in this present study, we aimed to investigate the prevalence of physical frailty and its associated factors among older people living in nursing homes in China.

## Methods

### Study design and participants

**Fig. 1:**
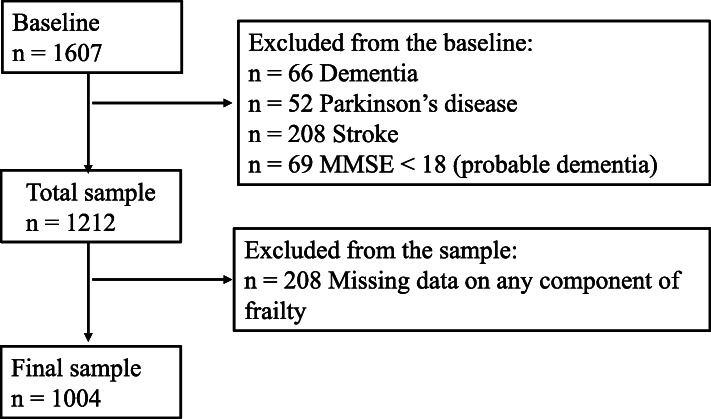
Assembly of the study sample

This cross-sectional study was conducted between January 2018 and April 2019 and involved 20 nursing homes in the metropolitan area of Changsha, China. A total of 2630 adults, aged ≥60, were invited to participate in this study. Changsha is a middle-size provincial city with a population of 8 million in the central region of China, which has the characteristics of low population mobility and a traditional Chinese lifestyle [20]. The survey comprised questionnaires, anthropometric measurements, and physical fitness tests, which were conducted according to the standard protocols of the study assessment tools. The questionnaire survey was completed via face-to-face interviews by well-trained nurses. In this study, inclusion criteria were as follows: having clear consciousness identified by registered nurses in nursing homes and the ability to communicate independently, including people who were visually or hearing impaired but could communicate through family members as identified by nursing home staff in each of the homes. We contacted all eligible participants by sending brochures explaining the study and inviting them to participate (*n* = 2550), and a total of 2204 residents agreed to participate in the survey, with a response rate of 86.4%. Of those, 1607 participants who completed the baseline survey by sending brochures were included in this study (597 died or moved out of those nursing homes during the survey period). We excluded participants who had a history of dementia (*n* = 66), Parkinson’s disease (*n* = 52), stroke (*n* = 208), or a Mini-Mental State Examination (MMSE) score < 18 (*n* = 69). Also, participants with missing data on any components of physical frailty were excluded (*n* = 208). Therefore, the final sample included 1004 participants (339 men and 665 women) (Fig. 1).

### Instruments

#### Physical frailty

Frailty was defined according to the phenotype of physical frailty [5], which consists of weakness, slowness, low level of physical activity, shrinking and exhaustion [5]. The operational definitions of each component are shown in Table 1. *Weakness* was measured in kilograms by a handgrip dynamometer (KD - WLJ; KonDak, China). Participants were required to perform the test twice for each hand in a standing position. The maximum value among the four measurements was used for the analyses. The cut-off points [21] were stratified by sex and body mass index (BMI). *Slowness* was defined as the average walk speed in a 5-m walking test. Starting from a motionless position, participants were instructed to take this test twice, and the time (seconds) of gait speed was recorded with a digital stopwatch between the 3 and 8 m in each trial. We measured the time taken (in seconds) to pass 8 m to calculate gait speed (m/s). The cut-off points [21] were slow gait speed as stratified by sex and standing height. We used the cut-off points [21] in weakness and slowness referring to the general older adults in the CHARLS cohort, which was previously conducted and validated by the population-based lowest quintile [5]. *Low level of physical activity* was measured with the Chinese version of the Physical Activity Scale for the Elderly (PASE) questionnaire [22]. The cut-off points were stratified by sex to collect physical activity scale data [23]. *Shrinking* was measured by unintentional weight loss > 5 kg during the previous year, except for dieting or exercise. *Exhaustion* was determined on the basis of a positive answer to either of the following two self-reported questions of the Center for Epidemiologic Studies-Depression (CES-D) Scale [24]: “I felt that everything I did was an effort” (in the past month) and “I could not get going.” According to the frailty phenotype [5], participants with three or more affected components were considered frail, those with one or two affected components were considered prefrail, and those without any component were considered robust.

**Table 1:** Operational definition of physical frailty phenotype

Frailty Phenotype	Measurement	Men	Women
Weakness	Grip strength [16] (stratified by sex and BMI [kg/m^2^], maximum value of either hand)	BMI ≤ 20.6: grip strength ≤25.2 kg BMI 20.6–23.2: grip strength ≤28.5 kg BMI 23.2–25.9: grip strength ≤30.0 kg BMI > 25.9: grip strength ≤30.0 kg	BMI ≤ 20.0: grip strength ≤15 kg BMI 20.0–22.1: grip strength ≤17.5 kg BMI 22.1–24.8: grip strength ≤17.8 kg BMI > 24.8: grip strength ≤20.0 kg
Slowness	Walking speed (5 m) [16] (stratified by sex, averaged value of two repeated measurements)	Height ≤ 163 cm: ≥ 0.90 m/s Height > 163 cm: ≥ 0.96 m/s	Height ≤ 151 cm: ≥ 0.72 m/s Height > 151 cm: ≥ 0.86 m/s
Low level of physical activity	Self-reported: Physical Activity Scale for the Elderly (9 items)	Score of physical activity per week < 56.4 points [18]	Score of physical activity per week < 58.8 points [18]
Shrinking	Unintentional weight loss	In the last year, self-report of losing more than 5 kg unintentionally (i.e., not due to dieting or exercise) or unintentional weight loss of at least 5% of body weight	
Exhaustion	Two items of the Center for Epidemiologic Studies-Depression Scale	a) I felt that everything I did was an effort. b) I could not get going. The question asked, “how often in the last week did you feel this way?” 0 = rarely or none of the time (< 1 day), 1 = some or a little of the time (1–2 days), 2 = a moderate amount of the time (3–4 days), or 3 = most of the time. Subjects answering “2” or “3” to either of these two questions were categorized as being exhausted.	
Overall frailty status	Non-frail: 0 affected criteria; pre-frail: 1–2 affected criterion; frail: ≥ 3 affected criterion.		

*BMI* Body mass index

#### Correlates of physical frailty

Information on the following covariates was collected through the questionnaire: age, sex, education level (elementary and below, or junior middle school and above), marital status (married, other [widowed, divorced, never married]), type of institution (public or private), living status (living with husband/wife, living with alone or others [unknown person]), current drinking (yes or no), current smoking (yes or no), regular exercise (≤ 2 times/week, ≥ 3 times/week), and self-reported health (very good or good, fair or poor). Basic activities of daily living (ADL), as the correlate of frailty, was measured by the Katz scale, which comprises including the following 6 items: bathing, dressing, toileting, transferring, continence, and feeding [25]. Disability in ADL ability was defined as having difficulty and/or requiring assistance in time ≥ 1 activities. Information on the medical history of diseases was collected from the medical records by physicians. Comorbidity was defined as having 2 or more of the following 9 diseases, hypertension, diabetes, cancer, chronic heart disease, stroke, chronic digestive disease, arthritis/rheumatism, chronic lung disease, and chronic kidney disease.

### Statistical analysis

The characteristics of the sample were summarized according to physical frailty groups. Categorical variables were presented as percentages. Trends in characteristics across physical frailty status were tested using the Cochran-Mantel-Haenszel test. Odds ratios (OR) with 95% confidence interval (CI) of physical frailty for the potential associated factors were estimated by using multinomial logistic regression models. The logistic regression model included age (continuous), women (reference men), low education level (reference junior middle school and above), being widowed or divorced or never married (reference being married), living in a private institution (reference public), living alone or with unknown person (reference living with husband/wife), current drinking (reference no), current smoking (reference no), regular exercise ≤2 times/week (reference regular exercise ≥3 times/week), and poor self-reported health (reference very good/good self-reported health). A Venn diagram was used to illustrate the overlap of ADL disability and comorbidity with physical frailty.

All statistical analyses were performed using IBM SPSS Statistics Version 24.0 (IBM; Armonk, NY, USA). All *p*-values were two-tailed, and *p* ≤ .05 was considered statistically significant.

## Results

Participants were 60 to 107 years of age, with mean age of 80.8 (standard deviation [SD] 8.9) years and 33.8% were men. The prevalence of physical frailty and prefrailty were 55.6 and 38.5% respectively. In men, 37.2% were pre-frail and 30.5% were frail; in women, 62.8% were pre-frail and 69.5% were frail. Table 2 shows characteristics of participants according to physical frailty status. A trend test of all factors across physical frailty status revealed that those participants who were more frail compared with participants who were less frail, tended to be older, more likely to be women, to be widowed or divorced or never married, to be living in a private institution, to be living with alone or others (unknown person), to report poor health status, and were less likely to have regular exercise.

**Table 2:** Characteristics of participants according to physical frailty status

	Overall	Frailty status			
		Robust (*n* = 59)	Prefrail (*n* = 387)	Frail (*n* = 558)	*P* value for trend
Age, mean (SD), years	80.8 (8.9)	70.9 (7.5)	79.6 (8.6)	82.7 (8.4)	< 0.001
Sex					0.01
Men, %	33.8	42.4	37.2	30.5	
Women, %	66.2	57.6	62.8	69.5	
Education levels					0.469
Elementary and below, %	51.1	57.6	50.9	50.5	
Junior middle school and above, %	48.9	42.4	49.1	49.5	
Marital status					< 0.001
Married, %	23.9	59.3	26.9	18.1	
Others (widowed, divorced, never married), %	76.1	40.7	73.1	81.9	
Type of institution					< 0.001
Public, %	55.5	78.0	63.0	47.8	
Private, %	44.5	22.0	37.0	52.2	
Living status					< 0.001
Living with husband/wife, %	23.8	61.0	27.1	17.6	
Living with alone or others (unknown person), %	76.2	39.0	72.9	82.4	
Current drinking (yes), %	24.8	30.5	20.4	27.2	0.217
Current smoking (yes), %	11.6	16.9	11.9	10.8	0.213
Regular exercise					< 0.001
≤ 2 times/week, %	28.4	11.9	22.5	34.2	
≥ 3 times/week, %	71.6	88.1	77.5	65.8	
Self-reported health					< 0.001
Very good or good, %	19.2	33.9	22.5	15.4	
Fair or poor, %	80.8	66.1	77.5	84.6	

Continuous variables are expressed as mean ± standard deviation; categorical variables are expressed as percentages

**Fig. 2:**
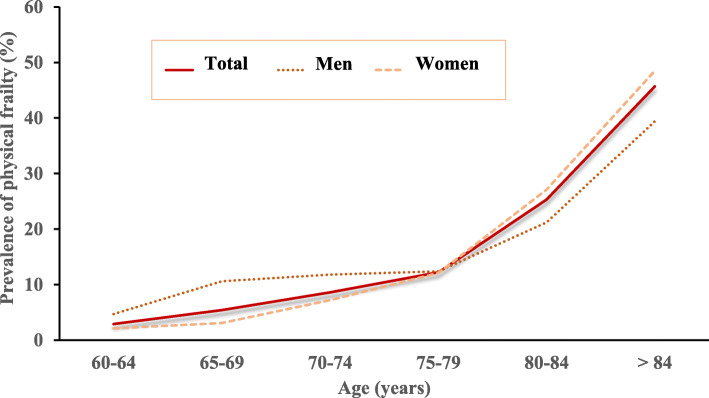
Estimated prevalence of physical frailty by sex and age

Figure 2 shows the prevalence of physical frailty by sex and age group. The prevalence of physical frailty increased with each successive 5-year age group (p for trend < 0.001). The increasing trend of the curve was similar in both men and women. The percentage of physical frailty dramatically increased from the 75–79 age group, especially in women.

**Fig. 3:**
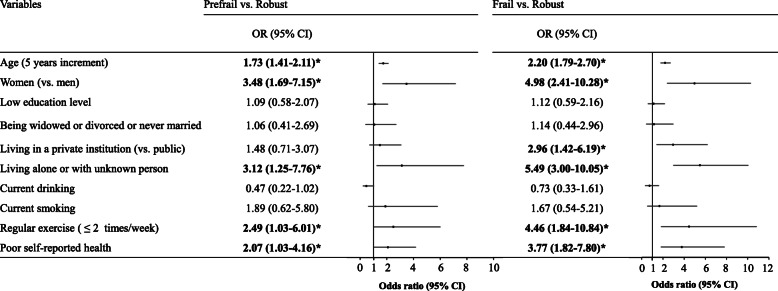
Multivariable-adjusted odds ratio and 95% confidence interval of physical frailty according to its potential associated factors using multinomial logistic regression model. Note: OR = Odds ratio, 95% CI = 95% confidence interval, *Significant association. OR (95% CI) of frailty status and its associated factors were estimated in multinomial logistic regression model for participants in the prefrailty or frailty vs those in robust

Figure 3 shows the results of multinomial logistic regression model on the associations of physical frailty with its potential risk factors. The multivariable-adjusted OR with each five-year increment in age was 2.20 (95% CI 1.79–2.70) for being frail and 1.73 (95% CI 1.41–2.11) for being prefrail compared to the robust group. Women were approximately 5 times more likely to be frail (multivariable-adjusted OR 4.98, 95% CI 2.41–10.28), and 3.5 times more likely to be prefrail (multivariable-adjusted OR 3.48, 95% CI 1.69–7.15) than men. Living alone or others (unknown person) was associated with significantly increased odds of frailty (multivariable-adjusted OR 5.49, 95% CI 3.00–10.05) and prefrailty (multivariable-adjusted OR 3.12, 95% CI 1.25–7.76). Compared with participants with regular exercise, those who were not engaged in regular exercise had a fourfold higher risk of being frail (multivariable-adjusted OR 4.46, 95% CI 1.84–10.84) and a twofold higher risk of being prefrail (multivariable-adjusted OR 2.49, 95% CI 1.03–6.01). Those who reported poorer self-reported health were approximately 4 times more likely to be frail (multivariable-adjusted OR 3.77, 95% CI 1.82–7.80) and 2 times more likely to be pre-frail (multivariable-adjusted OR 2.07, 95% CI 1.03–4.16). Living in private (vs. public) institutions was associated with increased odds of frailty (multivariable-adjusted OR 2.96, 95% CI 1.42–6.19), but not with prefrailty. No statistically significant associations were found between frailty status and education level, marital status, current drinking and current smoking.

**Fig. 4:**
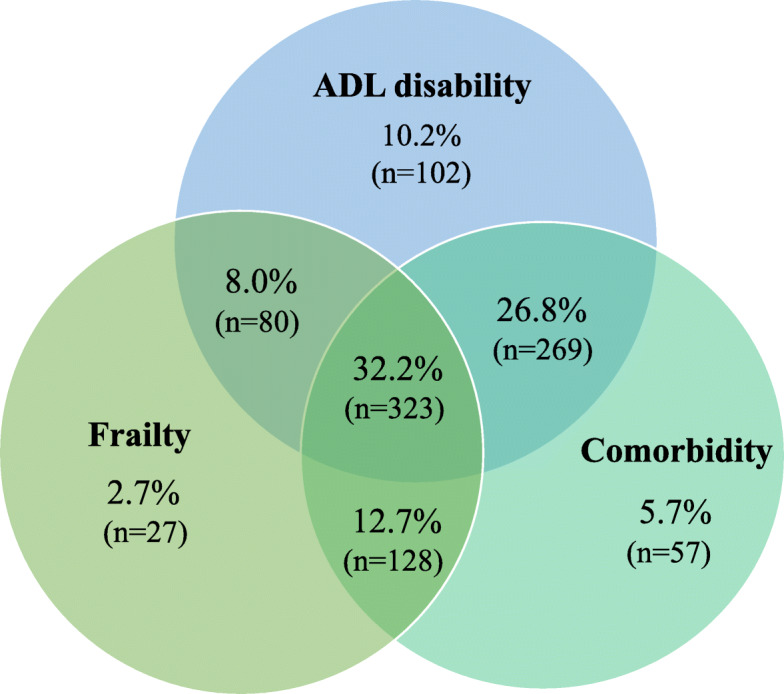
Venn diagram showing the extent of overlap of physical frailty with activity of daily living (ADL) disability and comorbidity. Among the overall 1004 participants, each participant had at least one of those three conditions. Of these, 558 were frail, 774 had ADL disability, and 777 had comorbidity. Disability: having difficulties in one or more ADL. Comorbidity: with 2 or more out of the following × chronic diseases: hypertension, diabetes, cancer, chronic heart disease, stroke, chronic digestive disease, arthritis/rheumatism, chronic lung disease, and chronic kidney disease

Figure 4 shows the overlap of ADL disability and comorbidity with frailty. Among the overall 1004 participants, each participant had at least one of those three conditions. Of these who were frail, 12.7% had comorbid diseases, 8.0% had ADL disability, 32.2% had both comorbid disease and ADL disability, and 2.7% had neither ADL disability nor comorbidity.

## Discussion

In this present study, our results showed that physical frailty was highly prevalent among older adults living in nursing homes in China, especially in women. We observed older age, being women, living in a private institution, living alone or others (unknown person), having no regular exercise, and poor self-reported health was significantly associated with physical frailty. Also, we found that although physical frailty, to some extent, overlapped with disability and comorbidity, many frail participants did not have disability or comorbidity, suggesting that physical frailty did not equate with comorbidity or disability in this study’s population of nursing home residents. At present, there is no the gold standard for comprehensive geriatric assessment as part of the admissions procedure to nursing homes in China. Most nursing in homes in North America and Europe use the Resident Assessment Instrument (RAI), data from which corresponds directly to the frailty index [26], however, such the RAI is currently not utilized in China.

Our preliminary study underlined the fact that as many as about one-half of the older adults living in nursing homes were frail, and 38.5% were still prefrail; moreover, frailty tended to be more prevalent in women than men. A number of studies [27–29] from different countries (i.e., Japan, Southern Italy) were consistent with our results, which demonstrated that frailty was highly prevalent, and the prevalence of frailty in women was higher than in men. A greater understanding of the reasons and implications of this physical phenotype across sex was required. An important reason suggested is that older women with frailty have more abdominal fat than older men [30]. Abdominal adiposity was associated with systemic inflammation by mediating its link with metabolic syndromes, which were important markers of oxidative stress and result in skeletal muscle damage and low grip strength [30]. This factor might be a core mechanism leading to sex-associated frailty. In our study of nursing homes in China, on average female residents were older than male residents and were better able to take care of themselves and others and to arrange their care. Hence, it would be interesting to look at who is admitted to a nursing home and reason for their admission. It could be that men are admitted with lower frailty levels when they live alone because they are less able to support or organize support for themselves [31]. Therefore, understanding the differences by sex in frail older adults might help us to shift towards more appropriate goal-directed approaches to improve the health status of males and females in different ways.

In agreement with some previous studies from other countries [10, 21, 32], we also observed that the prevalence of frailty increased with older age. In terms of the associations between physical frailty and status of the institution (private versus public), the present study was the first, to our knowledge, in which such an association was observed. One possible explanation could be that older adults living in private institutions (more expensive in the metropolitan areas than public institutions) usually have higher social economic status, are perhaps more likely to feel more lonely [13] and suffer loss of appetite [33] when staying away from family and changing their ways of previous life, resulting in higher likelihood of being frailer. Compared with people living with their partners in the same nursing home, those who live with alone or share the room with unknown person may become more frail because of poorer social ties [34] and mental disorders [35]. In line with previous studies [36, 37], we also found that regular exercise was associated with physical frailty. One explanation is that there is a vicious cycle: due to the fear of falling [38], as the level of frailty increases, so does the tendency to avoid taking regular exercise. Participants with frailty had poorer self-reported health in our study, in concordance with results from previous studies [14, 39, 40]. This might be explained by the fact that frail older people have a state of greater vulnerability [41], so they were more likely to rate their health poorly. In all, the potential role of those factors of physical frailty warrant further investigations to explore their clinical application among elderly nursing home residents.

Reduced physical function reserve (i.e., physical frailty) may occur without showing any difficulties in tasks of daily living or having multiple chronic diseases [5]. However, there are concerns as to whether physical frailty is synonymous to disability or comorbidity among older adults in nursing homes in China. This is because most older adults who moved to nursing homes are usually severely physically dependent and suffering from comorbidities [42–44] as older people usually live at home with their families, in keeping with the traditional Chinese family values [45, 46]. In the present study, in contrast, we found that many frail participants did not have disability or comorbidity, indicating that physical frailty did not equate with comorbidity or disability in the population of elderly nursing home residents. Our findings suggest that, physical frailty, related but distinct from disability and comorbidity, can be integrated into nursing-home settings as part of risk stratification and may serve a useful target for preventive interventions.

Our study had several strengths. Firstly, weakness and slowness for defining physical frailty were recorded using objective measures. Secondly, this study comprehensively reports examined substantial sociodemographic differences in physical frailty prevalence. However, there were limitations to our study. Firstly, due to the cross-sectional study design, there was no follow up to observe the progression from pre-frailty to frailty, and we could not establish causality of frailty and adverse health outcomes. Therefore, further research will be needed to verify the temporality of the exposure-effect association. Secondly, because of the relatively low response rate, there is a possibility of selection bias might have existed in our study. We could speculate that the nursing home residents who did not participate may be frailer. Thirdly, participants in our study were recruited from in one city, which is a capital city in the central region of China with a specific regional representation having low population mobility and a conventional Chinese lifestyle; therefore, caution should be practiced to generalize the findings of our study applicable to the whole of China. Finally, because most older adults in nursing homes were mostly 80 years and over, we might have underestimated frailty status.

## Conclusion

In conclusion, we demonstrated that physical frailty was highly prevalent among older adults living in nursing homes in China, especially in women. Older age, female sex, living in a private institution, living alone or with an unknown person, having no regular exercise, and poor self-reported health are significantly associated with physical frailty among elderly nursing home residents. Hence, given the reversible progression of frailty phenotype from pre-frailty to frailty, our findings should encourage the integration of physical frailty into nursing-home settings as part of risk stratification and as a useful target for preventive interventions. The potential role of those factors of physical frailty warrant further investigations to explore their clinical application among elderly nursing home residents.

## Data Availability

The datasets used for the current study are available from the corresponding author upon reasonable request.

## Abbreviations

BMI: Body mass index
OR: Odds ratio
95% CI: 95% confidence interval
SD: Standard deviation
ADL: Activity of daily living
MMSE: Mini-Mental State Examination
PASE: Physical Activity Scale for the Elderly
CES-D: The Center for Epidemiologic Studies-Depression
